# Copy Number Alterations and Methylation in Ewing's Sarcoma

**DOI:** 10.1155/2011/362173

**Published:** 2011-03-15

**Authors:** Mona S. Jahromi, Kevin B. Jones, Joshua D. Schiffman

**Affiliations:** ^1^Department of Oncological Sciences, Huntsman Cancer Institute, University of Utah School of Medicine, 2000 Circle of Hope, Salt Lake City, UT 84112, USA; ^2^Department of Orthopaedics, Huntsman Cancer Institute, University of Utah School of Medicine, 2000 Circle of Hope, Salt Lake City, UT 84112, USA; ^3^Center for Children's Cancer Research (C3R), Huntsman Cancer Institute, University of Utah School of Medicine, 2000 Circle of Hope, Salt Lake City, UT 84112, USA; ^4^Division of Pediatric Hematology/Oncology, Huntsman Cancer Institute, University of Utah School of Medicine, 2000 Circle of Hope, Salt Lake City, UT 84112, USA

## Abstract

Ewing's sarcoma is the second most common bone malignancy affecting children and young adults. The prognosis is especially poor in metastatic or relapsed disease. The cell of origin remains elusive, but the EWS-FLI1 fusion oncoprotein is present in the majority of cases. The understanding of the molecular basis of Ewing's sarcoma continues to progress slowly. EWS-FLI1 affects gene expression, but other factors must also be at work such as mutations, gene copy number alterations, and promoter methylation. This paper explores in depth two molecular aspects of Ewing's sarcoma: copy number alterations (CNAs) and methylation. While CNAs consistently have been reported in Ewing's sarcoma, their clinical significance has been variable, most likely due to small sample size and tumor heterogeneity. Methylation is thought to be important in oncogenesis and balanced karyotype cancers such as Ewing's, yet it has received only minimal attention in prior studies. Future CNA and methylation studies will help to understand the molecular basis of this disease.

## 1. Introduction

Ewing's sarcoma is a highly malignant tumor of children and young adults. The molecular mechanisms that underlie Ewing's sarcoma development are beginning to be understood, but the genetic risk factors leading to disease susceptibility remain largely unknown. Ewing's sarcoma is the second most common pediatric bone cancer after osteosarcoma, with 30–60% survival depending on tumor site and metastases at diagnosis [1, 2]. When patients with Ewing's sarcoma relapse, it is usually fatal: less than 20% survive [3–5]. Beyond incremental improvements in cytotoxic chemotherapy regimens, there have been no major treatment advances in the last 20 years [6, 7]. Clinical features are the only markers that have been found to correlate reliably with the outcome in Ewing's sarcoma, but no risk-adapted therapy has proven successful; worse prognosis in Ewing's is predicted by metastatic disease measured by imaging and bone marrow examination, larger tumor volume, and primary tumors in the pelvis [8]. While osteosarcoma is thought to originate from bone cell progenitors [9], the cell of origin of Ewing's sarcoma is less clear with some evidence suggesting that tumors arise from a mesenchymal stem or progenitor cells [10–12]. Other researchers in the field believe instead that Ewing's sarcoma develops from a neuroectodermal origin [13–17]. The lack of a known cell of origin contributes to the difficulty in understanding exactly how Ewing's sarcoma develops or even how to design laboratory experiments to study tumorigenesis.

Nearly every case of Ewing's sarcoma contains a translocation involving the *EWSR1* gene on chromosome 22. The most common rearrangement is t(11;22)(q24;q12), which generates the *EWS-FLI1* fusion oncogene, found in ~85% of Ewing's cases [18–21]. The translocation t(21;22)(q22;q12) is found in another 10% of cases [21, 22] and the remainder of EWS translocations utilize a variety of fusion partners from the ETS family of transcription factors [19, 20]. All of the Ewing's sarcoma fusion proteins contain a strong transcriptional activation domain fused to a DNA-binding domain and function as aberrant transcription factors that dysregulate a number of target genes and contribute to oncogenic transformation [18–21, 23–30]. The EWS-FLI1 translocation is the best understood and most well-characterized molecular aspect of Ewing's sarcoma. This translocation (or one of the alternates) is thought to be necessary but not sufficient to cause disease [31]. 

 In addition to translocations, neoplastic development in cancer depends on other acquired molecular changes. Such changes in tumor biology include copy number alterations (CNAs), such as genomic deletions or amplifications, and methylation abnormalities. As newer technology has become available in recent years, we have learned more about CNAs and methylation in Ewing's sarcoma and possible associations with outcome, disease classification, and tumorigenesis. These molecular investigations have been limited by the rarity of Ewing's sarcoma and the small tumor samples obtained at initial diagnostic biopsy available for analysis. Nevertheless, many overlapping regions of CNAs and methylation have been described; their underlying significance is not always clear. Further exploration as to how these changes affect the outcome and their prevalence is essential to the development of future treatment options. In this paper, we describe the reported CNAs and methylation changes associated with Ewing's sarcoma and any known clinical correlations with these molecular findings. 

## 2. Materials and Methods

Literature searches for articles containing “Ewing's sarcoma copy number” and “Ewing's sarcoma methylation” were performed via the PUBMED database. Results consisted of 15 separate journal articles for copy number and 14 for methylation. Twelve relevant publications were selected for copy number and their references explored and included when appropriate. Nine relevant promoter methylation articles were selected and their references explored. 

## 3. Results and Discussion

### 3.1. Ewing's Sarcoma and Copy Number Alterations (CNAs)

Specific CNAs predict prognosis in several cancers and have been introduced as part of clinical risk stratification for colorectal and breast cancer, neuroblastoma, and brain tumors [32–35]. Despite intense investigation of Ewing's sarcoma biology, very few molecular markers have been discovered for routine clinical use in this disease. In contrast, active risk stratification based on molecular cytogenetics has increased the cure rate for childhood acute lymphoblastic leukemia (ALL) from less than 50% to over 85% in only a few decades [36]. Moreover, the use of high-resolution single nucleotide polymporphism (SNP) technology has been used to identify recurring CNAs in childhood leukemia [37–42] including relapsed cohorts [43, 44]. The study of CNAs in cancer also helps to better classify and understand the development of disease. For example, *CDKN2A* homozygous deletions in pediatric gliomas were recently found to significantly associate with specific BRAF^V600E^ mutations, helping to define a new subset of tumors [45]; the same deletion and mutation were also shown to work together to promote glioma formation in mice, validating the cooperation between CNAs and mutations [46].

New genomic technology has proven effective in determining copy number changes in a variety of tissue types. Previously, DNA extracted from paraffin has been too degraded to yield reliable data for analysis, but a new molecular inversion probe assay (OncoScan, Affymetrix, Santa Clara, CA) has been used successfully to identify copy number changes in formalin-fixed paraffin-embedded (FFPE) samples [65]. The ability to now interrogate FFPE samples allows the analysis of archival tissues and increases sample sizes for future Ewing's sarcoma studies. Copy number assessment in combination with clinical data could be used to identify CNAs in archival tissue and determine their link to the outcome in Ewing's sarcoma. Moving forward, candidate loci could be further studied in vitro or in pre-clinical animal models to determine their contribution to drug resistance and tumor progression. 

A large number of novel and recurrent secondary abnormalities in Ewing's tumors relating to copy number already have been discovered (see summary Table 1). The vast majority of copy number studies thus far in Ewing's sarcoma have been performed with comparative genomic hybridization (CGH) technology on either cell lines or primary samples. The most commonly reported CNAs in Ewing's sarcoma are trisomies of chromosomes 8 and 12 followed by the gain of 1q [47–49, 51–57, 66, 67]. Trisomy 8 is of particular interest as it occurs consistently and often in high frequency, being reported in >50% of cases in Ewing's sarcoma [47, 56, 57]. The oncogene, *MYC*, is thought to be a possible candidate driver of trisomy 8 as it was shown to bestow a selective advantage through nonfocal amplification when studied in undifferentiated soft tissue sarcomas [67]. In contrast to these findings, many studies have not found a statically significant link to survival outcomes in Ewing's sarcoma and trisomy 8 [47, 51, 54, 56]. Despite the lack of statistical significance, some evidence does suggest a link between trisomy 8 and worse outcome or worse overall survival. Values for 5-year distant disease-free survival (*P* = .16) and overall survival (*P* = .39) were not statistically significant, but the percentage of trisomy 8 was greater in both survival categories implicating a possible, though not statistically relevant, trend [54]. Focal amplifications in both the long and short arms of chromosome 8 (opposed to the entire trisomy) have been associated with clinical outcomes in Ewing's sarcoma. Specifically, Ozaki et al. reported that 8p amplifications occurred at higher frequency in relapsed cases compared to primary tumors (*P* = .04) [48]. They also found that combinations of CNAs, including 8q amplification in conjunction with chromosome 20 amplifications, were significant for worse cumulative overall survival rates (*P* = .0065) [48]. Savola et al. have proposed *WDR67* (8q24.13) and *GSDMD1* (8q24.3) as interesting candidate genes for tumorigenesis and progression as part of 8q amplification that warrant future investigation based on their integrated outcome analysis (*P* < .001 and *P* < .001, resp.) [53]. 

Trisomy 12 has been suggested to be linked to trisomy 8. While one study found that every case with trisomy 12 was combined with trisomy 8 [51], others state the these trisomies are independent events [57]. The frequency of trisomy 12 occurring with trisomy 8 is higher than trisomy 12 alone, but both events have been shown to occur independently [47, 57]. Copy number gains of 8 and/or 12 appear more frequently in local recurrences (83% of the time) compared to primary (47%) and metastatic (42%) lesions and are hypothesized to appear with increased frequency during tumor progression or after initial translocation [57]. Much like trisomy 8, trisomy 12 has conflicting information regarding its clinical significance. However, many studies seem to suggest that trisomy 12 or focal amplifications on chromosome 12, are more important than those for chromosome 8. Trisomy 12 correlates to adverse-event-free survival (*P* = .009) for individuals with localized disease [47]. Even though other reports of this trisomy show no statistical significance for overall survival (*P* = .67) [54], evidence to the contrary links aberrations on 12p and 12q to reduced overall survival by univariate analysis (*P* = .039 and *P* = .019) [48]. In one set of Ewing's tumors, the smallest region of shared amplification on chromosome 12 contained two known oncogenes, *ERBB3* and *CDK4* [53]. These genes may be indicative of the importance of trisomy 12 and its role in tumorigenesis.

Amplifications and trisomies involving chromosomes 8 and 12 have conflicting findings regarding clinical and statistical significance. This is due to either the lack of statistical power in small sample sizes or the variable nature of the disease. In either case, neither trisomy was shown to be associated with *improved* prognostic outcome. This contrasts with descriptions of chromosome 2, which Brisset et al. reported to correlate with localized tumors rather than metastatic disease (*P* = .02) [52]. However, again illustrating the variable nature of copy number studies in Ewing's sarcoma, Ozaki et al. described the association between gains of 2q and the reduction of overall survival (*P* = .022) [48]. Perhaps the gain in chromosome 2 (specifically 2q) correlates with the more unusual localized tumors that also lead to relapse. Larger studies will be needed to clarify the importance of this amplification. 

The gain of 1q is often reported with the loss of 16q. This is the presumed artifact of an unbalanced translocation in Ewing's sarcoma, der(16)t(1;16) [47, 48, 51, 52, 54, 55, 66, 68]. Though it is difficult to separate the translocation's downstream effects from the resulting CNA's impact, specific clinical factors were linked to 16q loss such as age at diagnosis >15 years and disseminated disease at diagnosis (*P* = .035 and *P* = .038, resp.) [47]. The gain of 1q and the loss of 16q in combination with chromosome 12 gain also demonstrated an increased frequency of them occurring together (*P* < .0001) [47]. The region of 1q gain, regardless of localized or disseminated disease, was determined to be significant for both adverse overall survival (localized disease *P* = .002; disseminated disease *P* = .029) and event-free survival (localized disease *P* = .018; disseminated disease *P* = .010) [47]. While 1q amplification showed no statistical significance in other studies, a high-level focal amplification was found at 1q21-q22 [51], two genes also reported in other sarcoma samples [69], *SPRR3* with 5 copies and *FLG* with 4 copies were affected [51]. Other suspected candidates in 1q21-22 locus include *CACY* and *CAPL*, both of which have been implicated in tumor progression and metastasis [51, 70]. 1q21-1q22 amplification has also been reported in other sarcomas [69, 71]. This more focal 1q gain lacked statistical significance but still suggested association with adverse distant disease-free survival and overall survival [54]. 

Similar to the pairing of CNAs of 1q gain and 16q loss, combined losses of 16q and 17p, resulting from another unbalanced translocation, have been described [48, 55].The loss of concomitant 16q and 17p has demonstrated lower overall survival (*P* = .0012) [48]. 17p loss may have its major impact by encompassing the loss of the well-known tumor suppressor, *TP53* [47, 64, 72]. In addition to *TP53 *deletion that is contained within 17p loss, mutation of *TP53* has been reported to show an association with poor chemoresponse and overall survival in Ewing's sarcoma (*P* = .03 and *P* < .001) [60].

Deletion of 9p21 encompassing *CDKN2A (p16-INK4a)* appeared in 10–73% of cases, including Ewing's sarcoma cell lines [50, 53, 59–62], with reported *homozygous deletions* in 8% [56] and 13% [60] of patient samples. This *CDKN2A* deletion was found to be a negative predictor of disease-specific survival (*P* = .001): 7 patients with this deletion all died of disease before 36 months, 2 of which had metastases at diagnosis [63]. The combination of *CDKN2A* deletion and *TP53* mutation was shown to be the most significant negative predictor of overall survival (*P* < .001) [60]. Our own experience has demonstrated the 9p21 deletion to be much more common in cell lines (80%) than clinical samples (5%) (unpublished). Current studies validating the prevalence and prognostic significance of *CDKN2A* deletions and TP53 mutations in Ewing's sarcoma are underway through the Children's Oncology Group (COG). 

### 3.2. Genomic Instability

Instability of cancer genomes leads to the accumulation of CNAs. Early findings showed no statistical link between total number of CNAs and worse outcome in Ewing's sarcoma [51]. However, later data indicated that unstable karyotypes with higher numbers of CNAs in Ewing's tumors may be a correlate with worse outcome [53, 55, 56, 58]. CNAs totaling above three had worse prognosis in relation to event-free and overall survival (*P* = .049 and *P* = .030) [53]. By clustering patients into two groups of genomic instability and stable genomes, prognostic significance was determined for overall survival via univariate and multivariate analysis (*P* = .017 and *P* = .034) [56]. The group with increased genomic instability contained a reduced percentage of patients to reach complete remission, specifically 64% versus 100% [56]. 

### 3.3. Copy Number Mitochondrial Data

Mitochondrial DNA (mtDNA) copy number changes have been associated with increased risk of certain cancers. To date, breast cancer and renal cell carcinoma both have been associated with an increase in mtDNA and a decrease in mtDNA, respectively [73–75]. The displacement-(D-) loop of mitochondrial DNA (mtDNA), a noncoding region comprised of 1,124 base pairs, is more prone to mutation. These mutations, in conjunction with quantitative mtDNA changes, have been linked to Ewing's sarcoma [75, 76]. The D-loop's increased mutation stems from its vulnerability to oxidative damage and reduced reparation capacity [75]. Decreased copy number of mtDNA is more often found in samples containing D-loop mutations (*P* = .04) and could be a result of the transcriptional and replicating functions of the D-loop [75]. While both D-loop mutations and reduced content of mtDNA are at higher instance in Ewing's sarcoma, the greatest statistical significance was determined to be between low mtDNA copy number and tumor metastasis as all of the metastases in the study contained low numbers of mtDNA (*P* = .029) [75]. 

### 3.4. Ewing's Sarcoma and Methylation Data

Of the vast array of oncogenic manipulations of gene expression achieved in malignant cells, not all arise from either random mutation or cytogenetic gains and losses resulting in CNAs. Methylation is an alternate method by which gene expression is changed in cancer cells [77]. Methylation is the addition of a methyl group, usually to the 5^'^ position of the cytosine pyrimidine ring, most importantly on cytosine residues contiguous to guanine residues, in what are called CpG islands. CpG sequences, in general, are relatively scarce in the human genome, as spontaneous mutation of the C to a T residue is especially common in the methylated state. Most remaining CpGs in the human genome are in the 5^'^ regulatory and promoter sequences of genes. 

Methylation of these promoter CpGs provides a cell-heritable means by which expression can be regulated. When a new zygote is formed, the cell is extensively demethylated. As cell division proceeds and eventually differentiation, methylation also proceeds, silencing certain genes no longer necessary along the cell's prescribed differentiation course. Methylation of promoter CpG islands affects transcription of the nearby gene via physical interruption of the binding of transcription factors and by encouraging binding of methyl-CpG-binding domain proteins. This recruits histone deacetylase and other chromatin-remodeling proteins, resulting in tight chromatin packaging of the locus and exclusion of transcriptional machinery. This silenced state of the gene is then passed on to daughter cells. Normal methylation is an important developmental program by which dangerous genes, such as viral sequences integrated into the human genome over generations, and early developmental genes can be silenced when necessary.

Cancer cells can have a variety of problems with methylation. Some powerful oncogenes from integrated viruses and developmental genes that engender a highly proliferative state are often demethylated in cancers, resulting in their aberrant and deleterious expression. In addition, the promoters of many tumor suppressor genes are over-methylated resulting in their silencing. Obviously, genomic sequencing or usual hybridization techniques will not detect promoter methylation or demethylation. These powerful epigenetic modifications of genes are only noted when specifically sought. While dedicated efforts are underway to understand methylation in many cancer types, such large scale efforts are lacking for the Ewing's sarcoma family of tumors. In fact, the relative absence of wild karyotype anomalies and rampant mutations argues that epigenetic modifications such as methylation may be a prominent mechanism of disease in sarcomas bearing balanced translocations such as Ewing's sarcoma. While methylation and epigenetics have only received limited attention in the literature thus far, they seem likely to be important genetic mechanisms for Ewing's sarcomagenesis and progression.

Two studies have assessed genetic alterations in the 9p21 locus in Ewing's sarcoma [64, 78]. One identified 4 tumors with homozygous deletion and 2 with promoter hypermethylation of *CDKN2A* among 26 tumors in total [64]. Two tumors had codeletion of *CDKN2B (p15-INK4b)* and 3 promoter hypermethylation of p15 [64]. The second study found 1 methylated, 1 point-mutated, and 2 homozygous deleted *CDKN2A* among 24 tumors, as well as 2 methylated and 2 homozygous deleted *CDKN2B* [78]. 

Another gene studied with respect to promoter methylation in Ewing's sarcoma is *RASSF1A*. One study interrogated *RASSF1A* along with *p16, MGMT, GSTP1, APC, DAPK, RAR*β*, CDH1,* and *CDH13 *and found only *MGMT* and *CDH1* promoters methylated in 1 of 8 (12.5%) Ewing's sarcoma tumor samples [79]. Failure to detect* CDKN2A* promoter methylation in this study can be reconciled with the results of the larger studies described above based simply on insufficient sample size. With respect to *RASSF1A*, these results are more difficult to reconcile with the high frequency of *RASSF1A* methylation in a previously published report [80]; Avigad et al. identified 21 of 31 (68%) patient samples and 1 of 4 (25%) cell lines with hemizygous promoter methylation and 2 of 4 (50%) cell lines with homozygous promoter methylation. This larger study also correlated reduced *RASSF1A* expression with promoter methylation in 12 tumors checked. Further, they demonstrated reexpression of *RASSF1A* in the 2 homozygous methylated cell lines, upon in vitro application of 5-aza-2′ deoxycytidine, a powerful demethylating agent.

Two studies have corroborated each other in identifying 1 of 4 (25%) and 9 of 41 (22%) Ewing's cell lines with reduced caspase 8 expression, secondary to promoter methylation [81, 82]. The larger of these studies went further to confirm this reduced or lost caspase 8 expression by promoter methylation as the mechanism by which the 9 cell lines evaded TRAIL-induced apoptosis [82]. They confirmed the absence of deletions in the caspase 8 gene, as well as the reexpression of caspase 8 upon 5-aza-2′ deoxycytidine administration. Reexpression restored TRAIL and cytotoxic chemotherapy-induced apoptosis in these cell lines. They further checked 20 primary Ewing's sarcoma tumor samples, where they identified the predominance of the methylated caspase 8 promoter in 13 cases.

Finally, 5-aza-2′ deoxycytidine-driven demethylation has been tested as a means of disrupting the transformed phenotype of Ewing's sarcoma cell lines [83]. Using a clonogenic assay, demethylation alone dropped clonogenicity by 20 percent. Synergistic with a panel of histone deacetylase inhibitors, effects of 80 to 90 percent disruption of clonogenicity were detected, in addition to the reexpression of tumor suppressors such as E-cadherin and *TSLC1*. 

## 4. Conclusions

In this paper, we focused primarily on copy number and methylation data. We also acknowledge the importance of other molecular changes potentially at work in Ewing's sarcomagenesis such as pleiotropic effects of the chromosomal translocation beyond creation of the specific fusion oncogene, somatic mutations in yet uninterrogated tumor suppressors, increased expression of oncogenes or oncogenic microRNAs, and other epigenetic mechanisms of expression regulation such as histone and chromatin packaging that could not be covered within the scope of this review. CNAs and methylation changes in Ewing's sarcoma, along with some of these yet unexplored genetic and epigenetic perturbations may be essential to Ewing's tumorigenesis as evidence suggests that the EWS-FLI1 translocation is necessary but not sufficient for Ewing's transformation in vitro [31]; CNAs and methylation changes may form some of the necessary second hits required for Ewing's sarcoma to develop. The complex cooperative relationships of these many molecular mechanisms of expression alteration have not been fully explored, and a full-system biology approach may prove to be informative in the field of Ewing's sarcoma. As explored in this review, isolated combinations of chromosomal gains and deletions already have begun to be described. Unfortunately, the results of the limited copy number studies are rarely in agreement likely due to poor statistical power in each small sample studied. In many instances, statistical significance cannot be determined, but trends still suggest that these CNAs have prognostic impact or contribute to genomic instability associated with worse outcome. 

The investigation of copy number in Ewing's sarcoma will continue to advance given the rapid acceleration of high-resolution genomic technology to interrogate clinical samples, including archived FFPE specimens. Discovery of *specific genes* (rather than larger chromosomal cytobands) associated with tumor development and outcome will extend rapidly as the coverage in new SNP microarray platforms continues to become more dense and whole genome sequencing becomes more affordable. Novel and recurrent CNAs have been reported to cover nearly the entire genome. The main copy number recurrences in Ewing's sarcoma included trisomies 8 and 12, along with 1q amplification. These findings were consistent throughout the majority of studies, despite the inability of many studies to find statistical significance related to treatment response, prognosis, outcome, or tendency to relapse. Although several recurring regions, such as, 16q deletions, have been repeatedly shown in different copy number studies to be associated with worse outcomes, these findings still await validation and incorporation into clinical trials. 

For methylation as a mechanism of sarcomagenesis, two prominent tumor suppressor loci, *CDKN2A* and *RASSF1A,* as well as one important apoptosis activator, *caspase 8, *have been implicated. Further, functional assays have shown the reversibility of these expression repressions by the application of demethylating agents. For these methylation-associated genetic perturbations, therapeutic implications are very direct because the clinical drugs affecting methylation status and downstream histone deacetylation are already available for patient use. We expect that researchers have only scratched the surface of the Ewing's methylome. With the knowledge of demethylated oncogenes and other methylation-silenced tumor suppressors, the mechanisms leading to further CNAs and increased genomic instability with tumor proliferation may be elucidated. The continued investigation of copy number and methylation in Ewing's sarcoma will lead to a better understanding of tumorigenesis, more accurate risk stratification and hopefully new targets for developmental therapeutics. As genomic technology continues to improve, CNA and methylation changes detected in clinical samples can be rapidly incorporated into patient care to improve the outcome in Ewing's sarcoma. 

## Figures and Tables

**Table 1:** Summary of copy number alternations (CNAs) in Ewing's Sarcoma (tumor, cell line, and xenograft) in published literature.

Deletion	Gain	Ewing's sample type	Frequency (%)	Technology	Study	Clinical significance
1p		ESFT	17/184 (9%)	Karyotyping and CGH	Hattinger et al. [47]; Ozaki et al. [48]	
1p36		ESFT	5/88 (6%)	Karyotyping (G-Band)	Roberts et al. [49]	
1p36.32-p36.11		ESFT	2/9 (22%)	SNP Microarray (Affy 100 K)	Neale et al. [50]	
	1q	ESFT	77/396 (19%)	Karyotyping and CGH	Armengol et al. [51]; Brisset et al. [52]; Hattinger et al. [47]; Ozaki et al. [48]; Roberts et al. [49]; Savola et al. [53]; Tarkkanen et al. [54]	(i) Adverse event free survival (ii) Adverse overall survival (iii) Age at diagnosis >15 years (iv) Metastatic (trend)
		Cell line	5/8 (63%)	CGH	Shing et al. [55]	
	1q21-q22	ESFT	5/28 (18%)	CGH	Tarkkanen et al. [54]	(i) Adverse overall survival (trend) (ii) Adverse 5-year distant disease-free survival (trend)

	2	ESFT	38/262 (15%)	Karyotyping and CGH	Brisset et al. [52]; Hattinger et al. [47]; Roberts et al. [49], Savola et al. [53]	(i) Localized disease
	2q	ESFT	12/62 (19%)	CGH	Ozaki et al. [48]	(i) Adverse overall survival

3p		Cell line	3/8 (38%)	CGH	Shing et al. [55]	

	4p	ESFT	10/105 (10%)	CGH	Brisset et al. [52]; Ozaki et al. [48]	(i) Relapse

	5	ESFT	28/231 (12%)	Karyotyping and CGH	Brisset et al. [52]; Hattinger et al. [47]; Roberts et al. [49]	
	5p	ESFT	5/25 (20%)	CGH	Ferreira et al. [56]	

	6p21.1~pter	ESFT	3/28 (11%)	CGH	Tarkkanen et al. [54]	(i) Adverse overall survival (ii) Adverse 5-year distant disease-free survival

	7	ESFT	26/216 (12%)	Karyotyping and CGH	Hattinger et al. [47]; Roberts et al. [49]; Tarkkanen et al. [54]	
	7p21.1-p11.2	ESFT	2/9 (22%)	SNP Microarray (Affy 100 K)	Neale et al. [50]	
7q (partial)		ESFT	6/25 (25%)	CGH	Ferreira et al. [56]	
	7q	ESFT	5/28 (18%)	CGH	Tarkkanen et al. [54]	

	8	ESFT	197/413 (48%)	Karyotyping, CGH and FISH	Armengol et al. [51]; Brisset et al. [52]; Ferreira et al. [56]; Hattinger et al. [47]; Maurici et al. [57]; Ozaki et al. [48]; Savola et al. [53]; Tarkkanen et al. [54]; Zielenska et al. [58]	(i) Local recurrences (trend) (ii) Relapse (trend) (iii) Adverse overall survival (trend) (iv) Adverse 5-year distant disease-free survival (trend)
		Cell line	8/8 (100%)	CGH	Shing et al. [55]	
	8p	ESFT	30/62 (48%)	CGH	Ozaki et al. [48]	(i) Relapse
	8q	ESFT	32/62 (52%)	CGH	Ozaki et al. [48]	
	8q11.21-q22.3	ESFT	6/9 (67%)	SNP Microarray (Affy 100 K)	Neale et al. [50]	
	8q24.11-q24.21	ESFT	7/9 (78%)	SNP Microarray (Affy 100 K)	Neale et al. [50]	

9p		ESFT	7/31 (23%)	CGH	Savola et al. [53]	
9p21		ESFT	50/291 (17%)	Karyotyping, CGH, FISH, Southern Blot, SNP Microarray (Affy 100 K), and MLPA	Brownhill et al. [59]; Huang et al. [60]; Kovar et al. [61]; Neale et al. [50]; Roberts et al. [49]; Savola et al. [62]; Wei et al. [63]	(i) Adverse event free survival (trend) (ii) Adverse overall survival(iii) Axial(iv) progressive disease (trend) (v) Poor chemoresponse
9p21		Cell line	24/43 (56%)	CGH (Agilent 44 K and 244 K), Taqman qRT-PCR, FISH, Southern Blot and MLPA	Brownhill et al. [59]; Kovar et al. [61]; Savola et al. [62]	
9p21.3		Xenotransplant	4/12 (33%)	dPCR, FISH	López-Guerrero et al. [64]	

10		ESFT	12/87 (14%)	CGH	Ferreira et al. [56]; Ozaki et al. [48]	

	11p	ESFT	2/62 (3%)		Ozaki et al. [48]	(i) Relapse
	11q	ESFT	2/62 (3%)		Ozaki et al. [48]	(i) Relapse

	12	ESFT	104/434 (24%)	Karyotyping, CGH and FISH	Armengol et al. [51]; Brisset et al [52]; Ferreira et al. [56]; Hattinger et al. [47]; Maurici et al. [57]; Roberts et al. [49]; Savola et al. [53]; Tarkkanen et al. [54]; Zielenska et al. [58]	(i) Adverse event free survival (ii) Adverse overall survival(iii) Relapse (trend)
	12p	ESFT	12/62 (19%)	CGH	Ozaki et al. [48]	(i) Adverse overall survival
	12q	ESFT Cell line	11/62 (18%) 6/8 (75%)	CGH CGH	Ozaki et al. [48]Shing et al. [55]	(i) Adverse overall survival
	12q14.1-q15	ESFT	2/9 (22%)	SNP Microarray (Affy 100 K)	Neale et al. [50]	

	14	ESFT	11/143 (8%)	Karyotyping and CGH	Brisset et al. [52]; Hattinger et al. [47]	
	14q11.2	ESFT	2/9 (22%)	SNP Microarray (Affy 100 K)	Neale et al. [50]	

	15	ESFT	4/43 (9%)	CGH	Brisset et al. [52]	

	16p	ESFT	2/28 (7%)	CGH	Tarkkanen et al. [54]	
16q		ESFT	69/396 (17%)	Karyotyping and CGH	Brisset et al. [52]; Ferreira et al. [56]; Hattinger et al. [47]; Ozaki et al. [48]; Roberts et al. [49]; Savola et al. [53]; Tarkkanen et al. [54]	(i) Adverse overall survival(ii) Age at diagnosis >15 years(iii) Disseminated disease at diagnosis
16q		Cell line	5/8 (63%)	CGH	Shing et al. [55]	
16q22.3		ESFT	5/9 (56%)	SNP Microarray (Affy 100 K)	Neale et al. [50]	

17		ESFT and Xenotransplant	2/19 (11%)	dPCR, FISH	López-Guerrero et al. [64]	
17p		ESFT Cell line	9/62 (15%) 4/8 (50%)	CGH CGH	Ozaki et al. [48] Shing et al. [55]	(i) Adverse overall survival
17p13		ESFT	8/88 (9%)	Karyotyping (G-Band)	Roberts et al. [49]	
	17q21.31-q25.3	ESFT	6/9 (67%)	SNP Microarray (Affy 100 K)	Neale et al. [50]	

	18	ESFT	6/68 (9%)	CGH	Brisset et al. [52]; Ferreira et al. [56]	

19		ESFT	4/25 (16%)	CGH	Ferreira et al. [56]	
19p		ESFT	7/62 (11%)	CGH	Ozaki et al. [48]	
19q		ESFT	11/62 (18%)	CGH	Ozaki et al. [48]	

	20	ESFT	35/248 (14%)	Karyotyping and CGH	Brisset et al. [52]; Ferreira et al. [56]; Hattinger et al. [47]; Roberts et al. [49]	(i) Adverse event free survival (ii) Adverse overall survival
	20p	ESFT	11/62 (18%)	CGH	Ozaki et al. [48]	(i) Adverse overall survival
	20q	ESFT	11/62 (18%)	CGH	Ozaki et al. [48]	(i) Adverse overall survival
	20q11.23-q13.33	ESFT	2/9 (22%)	SNP Microarray (Affy 100 K)	Neale et al. [50]	

	21q22.3	ESFT	2/9 (22%)	SNP Microarray (Affy 100 K)	Neale et al. [50]	
	22q11.21	ESFT	2/9 (22%)	SNP Microarray (Affy 100 K)	Neale et al. [50]	

Y		Cell lines	3/5 (60%)	CGH	Shing et al. [55]	

*Modified from Toomey et al. *Oncogene* 2010. ESFT: Ewing's Sarcoma Family of Tumors, CGH: comparative genomic hybridization.
